# Discovery of Brassica Yellows Virus and Porcine Reproductive and Respiratory Syndrome Virus in *Diaphorina citri* and Changes in Virome Due to Infection with ‘*Ca*. L. asiaticus’

**DOI:** 10.1128/spectrum.04996-22

**Published:** 2023-03-21

**Authors:** Jinming Lu, Lixia Zeng, Paul Holford, George A. C. Beattie, Yanjing Wang

**Affiliations:** a College of Forestry and Biotechnology, Zhejiang A&F University, Linan, Hangzhou, Zhejiang, China; b College of Plant Protection, South China Agricultural University, Guangzhou, Guangdong, China; c School of Science, Western Sydney University, Penrith, New South Wales, Australia; d The Key Laboratory for Quality Improvement of Agricultural Products of Zhejiang Province, College of Advanced Agricultural Sciences, Zhejiang A&F University, Linan, Hangzhou, Zhejiang, China; South China Agricultural University

**Keywords:** Asian citrus psyllid, virus, brassica yellows virus, porcine reproductive and respiratory syndrome, metatranscriptomics

## Abstract

Detection of new viruses or new virus hosts is essential for the protection of economically important agroecosystems and human health. Increasingly, metatranscriptomic data are being used to facilitate this process. Such data were obtained from adult Asian citrus psyllids (ACP) (*Diaphorina citri* Kuwayama) that fed solely on mandarin (*Citrus* ×*aurantium* L.) plants grafted with buds infected with ‘*Candidatus* Liberibacter asiaticus’ (*C*Las), a phloem-limited bacterium associated with the severe Asian variant of huanglongbing (HLB), the most destructive disease of citrus. Brassica yellows virus (BrYV), the causative agent of yellowing or leafroll symptoms in brassicaceous plants, and its associated RNA (named as BrYVaRNA) were detected in ACP. In addition, the porcine reproductive and respiratory syndrome virus (PRRSV), which affects pigs and is economically important to pig production, was also found in ACP. These viruses were not detected in insects feeding on plants grafted with *C*Las-free buds. Changes in the concentrations of insect-specific viruses within the psyllid were caused by coinfection with *C*Las.

**IMPORTANCE** The cross transmission of pathogenic viruses between different farming systems or plant communities is a major threat to plants and animals and, potentially, human health. The use of metagenomics is an effective approach to discover viruses and vectors. Here, we collected buds from the *C*Las-infected and *C*Las-free mandarin (*Citrus* ×*aurantium* L. [Rutaceae: Aurantioideae: Aurantieae]) trees from a commercial orchard and grafted them onto *C*Las-free mandarin plants under laboratory conditions. Through metatranscriptome sequencing, we first identified the Asian citrus psyllids feeding on plants grafted with *C*Las-infected buds carried the plant pathogen, brassica yellows virus and its associated RNA, and the swine pathogen, porcine reproductive and respiratory syndrome virus. These discoveries indicate that both viruses can be transmitted by grafting and acquired by ACP from *C*Las+ mandarin seedlings.

## INTRODUCTION

Plants are infected with a diverse range of microbes that interact with their host and with each other. The majority of these microbes are not causal agents of disease (1), and many promote plant growth (2, 3) while others facilitate resistance to various plant pathogens (4). In addition to these endosymbiotic organisms, coinfections of multiple pathogens are common (5), and citrus trees in orchards are no exception often containing mixed infections of various pathogens, including viruses, many of which can be transmitted by arthropod vectors (6). The extent of these multiple infections and their interactions with each other and with their insect hosts is slowly being revealed due to the development of new molecular tools, especially next generation sequencing (7). After long-term, continuous interactions, once these viruses can colonize an insect’s gut, there is the possibility of phyllosphere transmission by insect vectors (8, 9).

Brassica yellows virus (BrYV) is a recently discovered virus that mainly infects cruciferous vegetables in which it causes mottling, yellowing, or leaf roll symptoms (10, 11). It infects a range of brassicaceous plants, including members of the genera *Brassica*, *Raphanus,* and *Sinapis* (12). BrYV was first identified in China in 2011 (10) and is tentatively placed in the genus *Polerovirus* (Solemoviridae); it has since been reported in Korea (11), Japan (13) and Australia (14). Molecular and phylogenetic studies show BrYV is closely related to turnip yellows virus (TuYV: Luteoviridae) (10, 14, 15), which has been reported throughout Europe, South Africa, China, Iran, Egypt, South Africa, Egypt, Morocco, and Australia, and infects crops and weeds, including members of the Brassicaceae, Fabaceae, Amaranthaceae and Asteraceae (see references in [14]). Poleroviruses are mostly transmitted by aphids in a circulative and nonreplicative mode (16–19); one known polerovirus is transmitted by whiteflies, Bemisia tabaci (Gennadius) (Hemiptera: Aleyrodidae) (20). A recent study showed that the expression of polerovirus proteins mediates changes in plant-aphid interactions and inhibits aphid induction of jasmonic acid signaling in plant hosts (21).

Porcine reproductive and respiratory syndrome was first recognized in the United States in 1987 and has become one of the most important diseases affecting the global pig industry (22, 23). The causative agent of the syndrome, porcine reproductive and respiratory syndrome virus (PRRSV: Nidoviridales: Arteriviridae), is a single-stranded, positive-sense RNA virus, and possesses a genome *ca*. 15 kb in length (24). Two genotypes of PRRSV can be found, the European (type 1) and North American (type 2) types that share only approximately 60% nucleotide similarity (25). The virus was first isolated in China in 1996 (26) and has evolved quickly and spread widely during the last 2 decades, causing large economic losses (27–29). To date, it has mainly been detected in domesticated pigs and wild boars (30). However, PRRSV can be acquired by mosquitoes (*Aedes vexans* (Meigen) (Diptera: Culicidae) and houseflies (Musca domestica L. [Diptera: Muscidae]) from infected pigs and in which it can be retained for a short period; the virus did not replicate within the insects (31–34). It can be mechanically transmitted by mosquitoes and houseflies, but the insects could not serve as biological vectors (31, 32).

*Diaphorina citri* Kuwayama (Asian citrus psyllid, ACP) (Hemiptera: Sternorrhyncha: Psyllidae) is an economically important pest of citrus. It is the major vector of the pathogenic bacterium ‘*Candidatus* Liberibacter asiaticus’ (*C*Las), which is the putative causal agent of the most severe form of huanglongbing (HLB), the most serious disease of citrus (35). Besides spreading HLB, ACP harbors phytopathogenic viruses such as *Citrus* tristeza virus (CTV: Closteroviridae) (36) and several putative insect-specific viruses, including *Diaphorina citri*-associated C virus (DcACV: family not assigned), *Diaphorina citri* bunyavirus (DcBV: Bunyaviridae), *Diaphorina citri* cimodo-like virus (DcCLV: Reoviridae), *Diaphorina citri* densovirus (DcDNV: Parvoviridae), *Diaphorina citri* flavi-like virus (DcFLV: Flaviviridae), *Diaphorina citri* picorna-like virus (DcPLV: Picorna-like virus), and *Diaphorina citri* reovirus (DcRV: Rheoviridae) (37–40).

Traditional viral screening often uses PCR and qPCR (38, 41), which depend on prior knowledge of the viral sequences. The technology of metatranscriptomics, based on high-throughput sequencing, can accurately and efficiently determine the RNA viromes present in tissue and environmental samples (42), including plants (43), mammals (44), and arthropods (39). In this study, metatranscriptomics and transmission electron microscopy were used to discover to uncover the composition of the viromes of ACP that may have been acquired from bud grafts taken from *C*Las+ and *C*Las– plants, to determine if the virome composition differs due to coinfection with *C*Las. Two viruses, the brassica yellows virus and the porcine reproductive and respiratory syndrome virus, are the first reports in ACP.

## RESULTS

**FIG 1:**
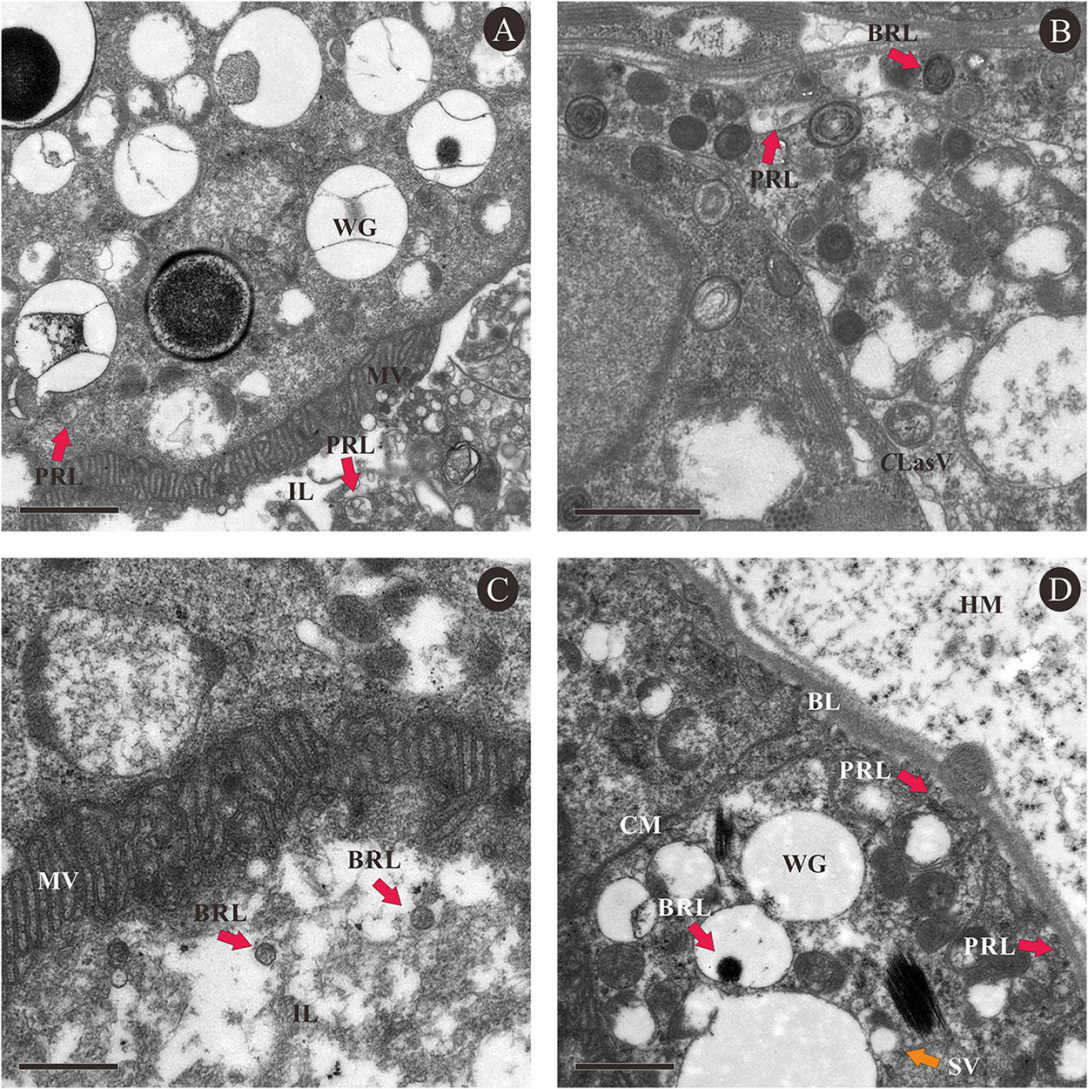
Transmission electron micrographs of cross section of gut tissues of *C*Las infected *D. citri*. A and B, cross section of filter chamber; C and D, cross section of midgut loop. Red arrows indicated viruses. MV, microvilli; WG, White secretory granule; ML, Muscle layer; MT, mitochondrion; IL, Intestinal lumen; CM, Cell membrane; HM, Hemocoel; SV, Secretory vesicle; BRL, BRYV-like; PRL, PRRSV-like; *C*LasV, *C*Las vertical section; *C*Las oblique section; *C*LasO, *C*Las oblique section; *C*LasC, *C*Las cross section. Scale bar 1000 nm.

TEM shows that viruses colonized in the filter chamber and midgut loop of the *C*Las+ ACP and passed into intestinal epithelial cells by incorporation of secretory granules (Fig. 1 A, D). Large accumulations of BrYV-like and PRRSV-like particles can be observed in the apical region close to microvilli (MV) in the intestinal lumen (Fig. 1C), close to the basal lamina (BL) (Fig. 1D), or between these locations (Fig. 1A, B). In addition, degradation of the intestinal microvilli was detected (Fig. 1A, C).

**FIG 2:**
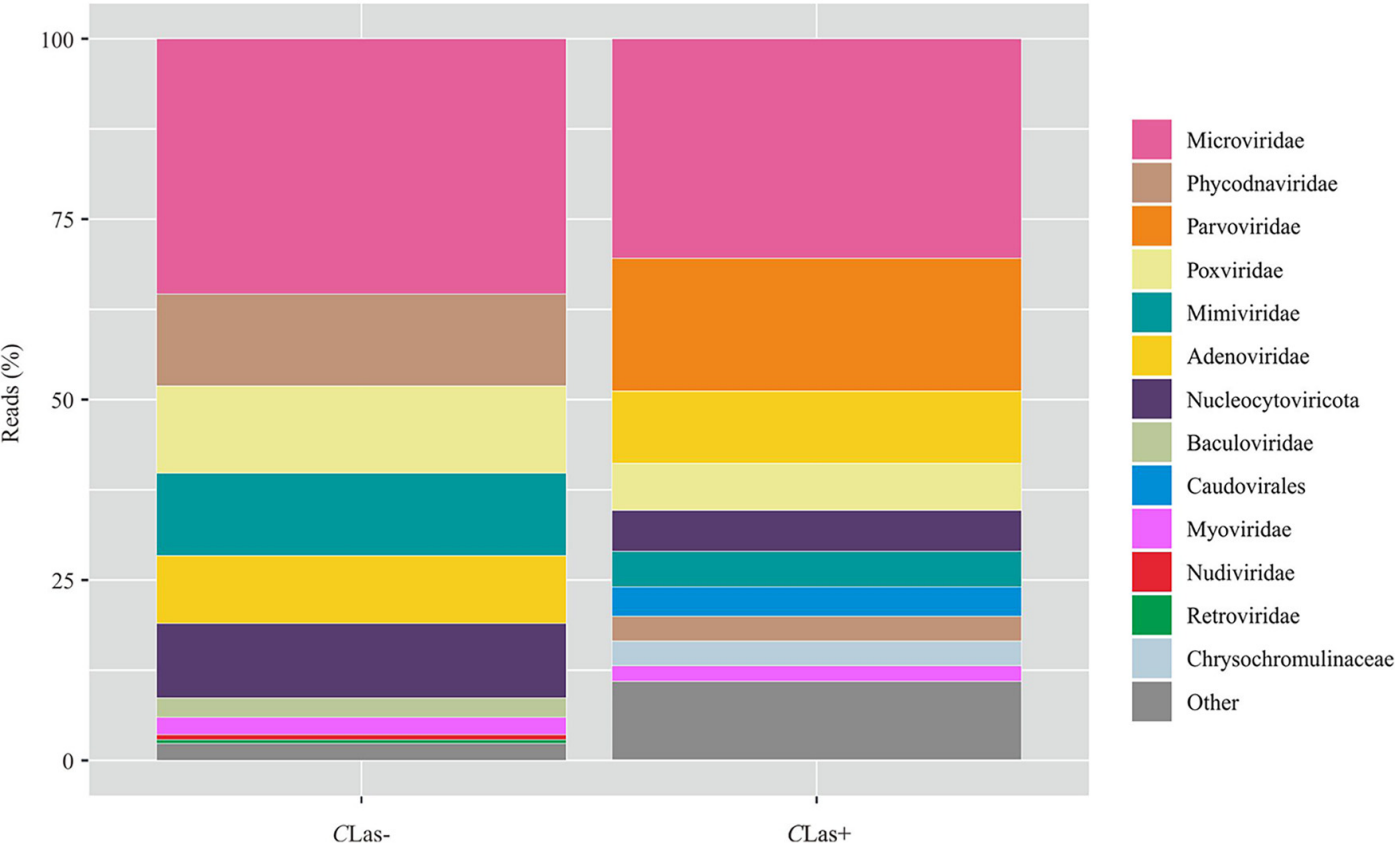
Viral composition of *C*Las-and CLas+ samples of *D. citri*. Relative proportions were determined by the number of reads classified to each virus clades using Kaiju.

A total of 38,676,951 and 34,843,908 clean reads were obtained from the *C*Las– and *C*Las+ samples, respectively. The presence of viral sequences from over 13 different viral families were identified from the Kaiju classification (Fig. 2) and a BLAST search, including DcDNV, DcPLV, DcRV, and citrus tristeza virus (CTV: Closteroviridae) (Table S1). The highest proportion of reads derived from both the *C*Las– and *C*Las+ samples came from the Microviridae. However, distinct differences in the proportions of reads from the other virus family were detected between the two sets of samples. Most notably, the *C*Las+ samples contained a high proportion of reads from the Parvoviridae and a smaller proportion from the Phycodnaviridae. In addition, two viruses, BrYV and porcine reproductive and PRRSV, were only detected in the *C*Las+ samples (Table S1).

### Putative insect-specific viruses.

First, DcDNV sequences were identified in *C*Las+ (2 contigs) and *C*Las– (3 contigs) samples of the Guangdong population. These sequences ranged from 326 to 2,339 nucleotides (nt) in length and showed >98% similarity to an isolate from a Taiwan population. Second, a BLAST search showed that contigs of 9,951 and 9,920 nt amplified from *C*Las+ and *C*Las– samples both showed 96.0% similarity to DcPLV found in an ACP population in Brazil. Lastly, a total of 111 contigs from *C*Las+ samples and 10 contigs from *C*Las– samples were identified as DcRV and showed >99% similarity to the DcRV-FL isolate recorded in Florida.

### Citrus tristeza virus.

The Kaiju classification and BLAST searches confirmed that 5 and 15 contigs from *C*Las+ and *C*Las– ACP samples, respectively, as CTV sequences. The contigs ranged from 260 to 3,997 nt in length and had a close relation to the CTV-FN08 isolate from Guangdong, China.

### Brassica yellows virus sequence.

**FIG 3:**
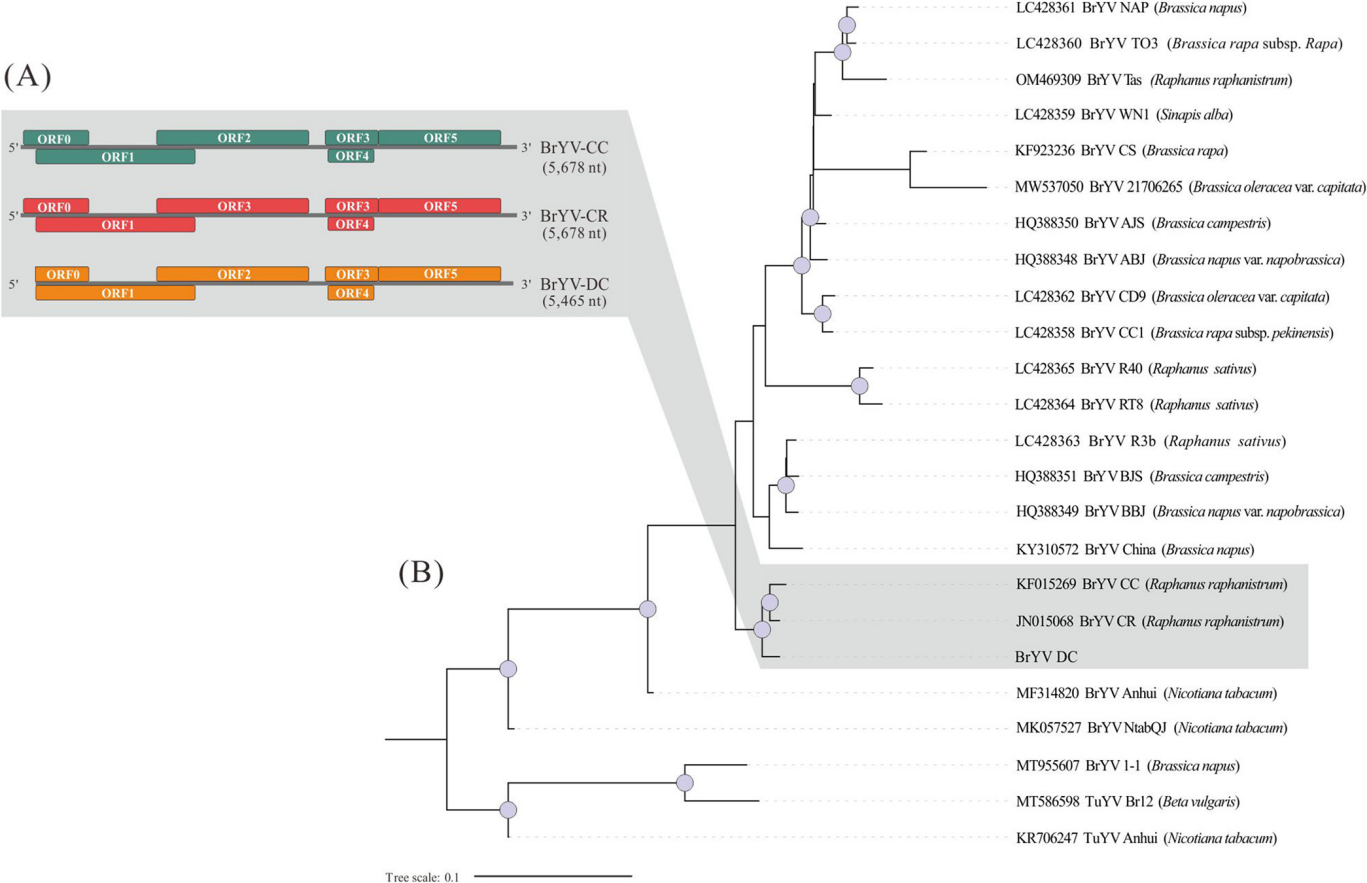
(A) Gene organization of BrYV-DC and its close related isolates; (B) Phylogenomic inference of BrYV using maximum likelihood method and a TIM2+F+R3 model. Circles on the nodes indicate bootstrap values ≥95.

A contig of 5,465 nt found in the *C*Las+ sample was named as BrYV-DC; this virus was not detected in the *C*Las– samples. It has the typical genomic organization of the BrYV genome, including 6 ORFs (ORF0–ORF5; Fig. 3A). The 5′ untranslated region (UTR) region was missing, while the 3′-UTR and ORF0 were incomplete.

To compare the new BrYV isolate obtained from ACP to previously characterized BrYV isolates (Table S2), a phylogenetic analysis using maximum likelihood (ML) was performed. The analysis showed most of the BrYV isolates being in the same monophyletic clade. BrYV-DC clustered with BrYV-CC and BrYV-CR, which were both associated with the plant host *Raphanus raphanistrum* L. (wild radish; Brassicales: Brassicaceae) (Fig. 3B).

**FIG 4:**
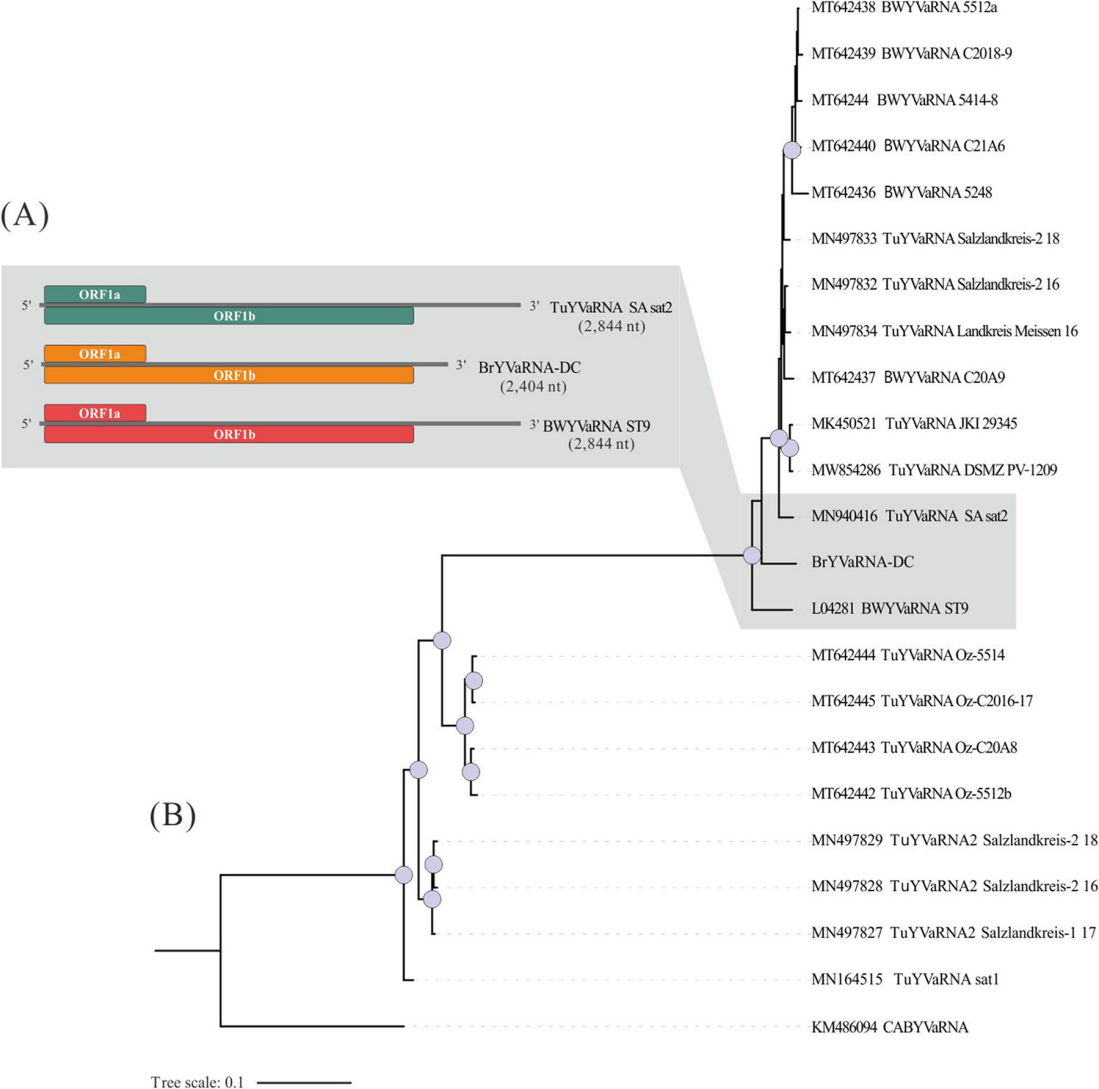
(A) Gene organization of BrYVaRNA-DC and its close related isolates; (B) Phylogenetic inference of virus-associated RNAs sequences using maximum likelihood method and a TVMe+R2 substitution model. Circles on the nodes indicate bootstrap values ≥95.

An additional contig of 2,404 nt was amplified from the *C*Las+ sample and shares 95.1% nt identity with a partial sequence of the Turnip yellows virus (TuYV)-associated RNA (TuYVaRNA; MN497833) and 94.9% nt identity with the complete sequence of beet western yellows virus (BWYV)-associated RNA (BWYVaRNA: KF533709). It has a typical genomic organization of Polerovirus-associated RNAs, encoding overlapping ORF1a and ORF1b (Fig. 4A). Compared with other TuYVaRNAs and BWYVaRNAs (Table S3), the contig from ACP forms a unique lineage in the phylogenetic tree (Fig. 4B). Thus, we propose the name “brassica yellows virus-associated RNA” (BrYVaRNA) for this newly identified RNA.

### Porcine reproductive and respiratory syndrome virus.

**FIG 5:**
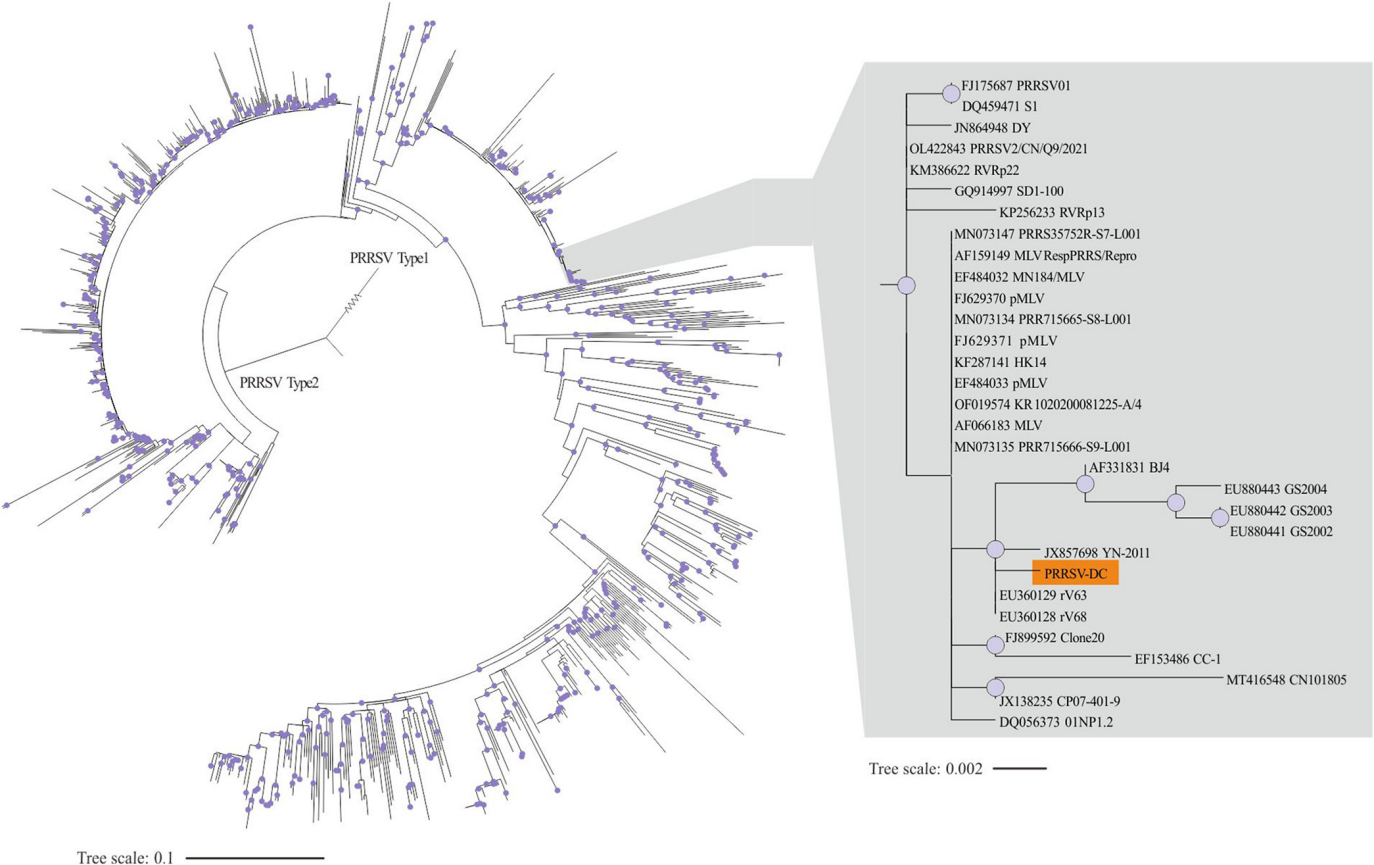
Phylogenetic inference of Porcine respiratory and reproductive syndrome virus (PRRSV) based on the ORF5 sequences using maximum likelihood method and a SYM+R6 substitution model. Circles on the nodes indicate bootstrap values ≥95.

A total of 11 contigs from the *C*Las+ sample (defined as PRRSV-DC in this study), ranging from 301 to 1,664 nt in length, were identified as PRRSV type 2 (Table S1). To infer the phylogenetic position of PRRSV-DC, we obtained 1,198 PRRSV type 2 and one PRRSV type 1 isolates from NCBI (Table S4). Based on the sequences of ORF5, phylogenetic analysis showed that PRRSV-DC belongs to the North American type (type 2) and grouped with isolates rV63, rV68, YN-2011, BJ4, GS2002, GS2003, and GS2004 (Fig. 5).

## DISCUSSION

### Brassica yellows virus sequences.

BrYV usually infects cruciferous crops; however, the most recent studies of this virus have found it in tobacco (Nicotiana tabacum L. [Solanales: Solanaceae]) (45) and strawberry (*Fragaria* ×*ananassa* Duchesne [Rosales: Rosaceae]) (46). In this study, we report a novel BrYV isolate associated with ACP that had fed on plants grafted with *C*Las+ buds. Our findings expand the insect and plant host range of BrYV; the virus has never been reported in members of the Rutaceae nor in ACP. In addition, this is the first report of the genomic sequence of BrYVaRNA in any plant; its original host plant(s) including orchard species and roadside weeds, need (s) to be determined. Some Polerovirus-associated RNAs cause severe of symptoms when in combination with a helper virus, e.g., BWYV ST9-associated RNA and Carrot red leaf virus-associated RNA (47). In addition, associated RNAs may help viruses avoid plant immunity and enhance infection. For example, the βC1 protein encoded by the satellite tomato yellow leaf curl China virus (TYLCCNV)-associated betasatellite (TYLCCNB) interacts with DEMETER in Arabidopsis thaliana (L.) Heynh. (Brassicales: Brassicaceae), which facilitates DNA glycosylase activity to decrease viral DNA methylation thereby promoting viral virulence (48). This is different from our previous understanding that viruses usually evolve in the direction of reduced pathogenicity. Therefore, the exploration of the mechanisms of BrYV transmission from its usual host is needed to facilitate crop biosecurity, particularly as host substitution often leads to virus mutation and the occurrence of new strains.

Our study suggests that BrYV can be transmitted by grafting, exist in *C*Las+ plants, and be acquired by ACP. The successful colonization of BrYV in mandarin in orchards is most likely related to transmission by other insect vectors—particularly phloem-feeding insects other than ACP—commonly found on citrus. There is also a possibility that organisms in the soil rhizosphere, phyllosphere fungi or microbial biofilms could be involved in transmission. For example, COVID-19 can be transmitted through biofilms, aerosols and wastewater (49–51). Moreover, the burning of biomass (straw and wood) in rural areas increases the black carbon, which increases the risk of COVID-19 spreading with aerosols (52). In addition, there is a strong fungal network in the rhizosphere of plants in the soil, which helps plants recruit microorganisms (53). Many viruses can be acquired by fungi and their spores (54). It has been shown that viral genomic sequences account for 0.02% of the citrus rhizosphere microbiome composition (55). The complex microbial community in the rhizosphere of plants can also protect plants from diseases (56). Whether BrYV has evolved to be transmitted by fungi needs further research. BrYV is transmitted by aphids (57) and five species, *Aphis* (*Toxoptera*) *aurantii* Boyer de Fonscolombe, *Aphis* (*Toxoptera*) *citricidus* (Kirkaldy), Aphis gossypii Glover, *Aphis spiraecola* Patch, and Myzus persicae (Sulzer) (Sternorrhyncha: Aphidae), associated with plant pathogens of citrus in China (58). Of these, Aphis gossypii is known to feed on *Brassica rapa* L. (syn. *B*. *campestris* L.) and from which it can transmit turnip mosaic virus and cucumber mosaic virus 2 (59). *Aphis spiraecola* has a broad host range, feeding on plants from over 90 families (60), and can spread a range of plant viruses (61). Myzus persicae feeds on more than 400 plant species from over than 40 families, including various brassica species and can transmit over 100 virus diseases (62, 63) including BWYV and four other poleroviruses (13). Hence, aphids, especially *M. persicae*, should be assessed as potential vectors of BrYV from infected nonrutaceous plants to citrus; however, other phloem-feeding insects that feed on citrus should also be examined.

Our results suggest that BrYV is able to colonize the filter chamber and midgut loop of ACP. Intestinal colonization implies the potential for persistent transmission, which plays a significant role in determining transmission of the virus (64); the acquisition and transmission of this virus by ACP needs further study. The virus can colonize *C. ×aurantium* and the intestinal channel of ACP together with *C*Las. Further exploration of interactions between BrYV and *C*Las is needed, as virus infection can affect plant resistance to other diseases. For example, the C4 protein of TLCYnV can inhibit mitogen-activated protein kinase (MAPK) cascades and the subsequent induction of defense responses (65). Four MAPK cascades genes (CsMAP9, CsMAP9-like, CsPR12, and CsPR14) are associated with *C*Las infection (66).

### Porcine reproductive and respiratory syndrome virus.

Our study showed that PRRSV can be transmitted by grafting and acquired by ACP from *C*Las+ mandarin seedlings. Thus, the virus not only survives in pigs and insects (31–34), but also survives in plants. There are few reports as to how animal viruses escape from plant immunity. In the present study, PRRSV was not detected in ACP that fed on plants grafted with *C*Las-budwood. Whether infection with *C*Las influences the colonization of host plants by viruses needs to be examined. How PRRSV entered the *C*Las+ plants was not determined, and this also requires further study. We propose three hypotheses with regard to co-colonization of citrus by *C*Las and PRRSV: (1) PRRSV can spread through a biofilm involving *C*Las in the crown and rhizosphere of plants; (2) *C*Las infection leads to increased susceptibility of citrus roots permitting entry via root damage caused by other organisms and (3) a boar or other unknown animals chewed the roots of citrus.

Biofilm production is a common trait associated with soil microorganisms (67)—viruses can attach to these structures (68) and gain protection from environmental stresses (69). PRRSV may persist in the soil due to interactions with biofilms. A species of *Chryseobacterium* was found to be the most abundant member of the rhizosphere microbiome of citrus plants (70, 71), many species of which have the ability to form biofilms and cause denitrification *in vitro* (72). Evidence for a role of biofilms in transmission comes from the fact that *C*Las can be successfully cultured with other microorganisms in a mixed-community biofilm with neutral to alkaline pH *in vitro* (pH 7.0 to 8.0) (70), and the biofilm may aid the persistence of both PRRSV and BrYV. The low concentration of *C*Las in biofilms indicates a strict dependence on the culture environment and the composition of associated bacteria (70). In the field, aerosol ammonium might facilitate biofilm formation through increasing pH—a large amounts of ammonia will be discharged around the pig farms (73). In addition, aerosols with particulate matter formed due to the burning of biomass increase the possibility of microorganisms that are adapted to neutral or alkaline humid environments (74). The complex interactivity between cloud feedback and aerosol-clouds may affect temperatures and humidity in orchards, which can be beneficial to the dispersal of viruses into plants mediated by plant transpiration (75). Furthermore, nitrogen deposition induces eutrophication and neutralizes the pH of the water, including groundwater and that in escape canals in the limestone mountains, which could increase the risk of microbial aggregation through bridging bacteria (74, 76). Due to the lack of nutrition data required by different plants at different growth stages in different climatic regions and the absence of biosensors, even intelligent water and fertilizer integration facilities often cause excessive water and fertilizer irrigation. Excessive irrigation not only wastes resource, but also provides a hotbed for pathogenic microorganisms colonization in the orchard depressions and ditches biofilm (77, 78). In addition, too much nitrogen in the soil often leads to acidification, which could be adjusted by *Chryseobacterium* colonization in citrus rhizosphere and might be beneficial to *C*Las colonization. Soil conditioners and compound fertilizers containing calcium carbonate, aluminum sulfate or iron sulfate used in groves will alter the pH of acidic soil. Additionally, calcium and magnesium ions can increase the ability of bridging bacteria to co-aggregate in biofilms in soil or wastewater (74). Hence, microbial biofilm formation in soil and groundwater might increase the risk of the co-transmission of PRRSV, BrYV and *C*Las. In addition, smelting and mining not only pollutes soil, causing heavy metal toxicity, but also increases the risk of transmission viruses and bacteria though reducing soil microbial diversity (79).

In the hypothesis of susceptibility caused by *C*Las, weakness of trees and poor immune balance of plants caused by *C*Las may lead to susceptibility to viruses. For example, sec-delivered effectors (SDE15) secreted by *C*Las can inhibit multiple members of papain-like cysteine proteases (PLCPs), which are extracellular broad-spectrum defense proteins (80, 81).

In China, pig farms are often located close to mountains, and orchards are often distributed in the mountains or at the foot of mountains; the orchard used in our study and a pig farm are located on a hillside. A route via the soil may be possible and facilitated by long-term fertilization with manure and mediated by root damage caused by soilborne pathogens. In addition, orchards are often attacked by wild boars or mice, which may also be a source of PRRSV.

Host substitution often leads to virus mutation and the occurrence of new strains. A partial genome was obtained in this study, with three situations: the virus is not fully adapted to the ACP and cannot replicate completely; the virus exists in ACP at a low concentration, suggesting that the virus cannot not replicate in insects (32, 33). The low concentration of the virus did not permit the *de novo* assembly of the virus, but the sequence data suggest parts are mutated which could lead to the formation of a new strain.

### Citrus tristeza virus.

Citrus tristeza virus was detected in the ACP populations feeding of plants grafted with both *C*Las+ and *C*Las– buds. It is probable, therefore, that this virus was widely distributed in the orchard from which the buds were sourced. Only a partial genome sequence of CTV was obtained from these two ACP populations, suggesting that the virus is maintained at a low titer. Our results show that BrYV and PRRSV could colonize in the filter chamber and midgut loop of ACP that carry CTV. In Florida, presence of CTV widespread in ACP sourced throughout the state (82). This study found a relatively low abundance of CTV sequences compared to those of DcACV, as was also found in this current study in China and that of Britt et al. (82) suggest that CTV is likely a part of the citrus phloem contents consumed during ACP-feeding. Various interactions occur between liberibacters and CTV within their plant hosts. In Florida, coinfection of *C*Las with five strains of CTV did not result in any synergistic effects within different citrus cultivars as judged by symptoms or accumulation of the pathogens (83). However, a study by Fu et al. (84) found that coinfection of sweet orange with a mild-strain of CTV gave limited protection against *C*Las whereas the interaction of a severe strain and *C*Las was synergistic. Working with the ‘*Ca*. L. africanus’ in South Africa, van Vuuren et al. (85) found that some protection against the African form of HLB was conferred by infection with CTV. Therefore, the interactions between different strains of CTV and *C*Las needs further study.

### Conclusion.

This study using metatranscriptomic data have shown differences the viromes of ACP that had fed on *C*Las– and *C*Las+ plants. In addition, this study is the first to find BrYV in ACP and to find its associated Polerovirus-associated RNAs, BrYVaRNA, in any host. The study also found PRRSV in the psyllid. The whole genome of BrYV and partial genome of PRRSV were *de novo* assembled. To determine the source of these viruses, further metagenomic studies should be undertaken that incorporate the plants, soils and insects that occur in and around the citrus grove. In addition, due to the potential for persistent transmission by insect vectors, transmission studies of psyllids infected with these viruses urgently need to be undertaken.

## MATERIALS AND METHODS

### Plant and insect material.

*C*Las-infected ‘Shatangju’ potted mandarin trees were collected from Boluo County (23°30′50.19″ N, 114°37′3.05″ E) in east-central Guangdong province, China, and buds from them were cut and grafted onto *C*Las–free mandarin plants. Buds from the *C*Las-free plants were also budded onto *C*Las–free mandarin plants to act as controls. All the grafted citrus plants were kept in a small, insect-proof net house. After 6 months, each plant was assayed by qPCR for the presence of *C*Las with the primer pair, HLBas/HLBr, targeting the 16S rRNA gene (86). The infected (*C*Las+) ‘Shatangju’ plants with CT values <25, and the uninfected (*C*Las-) control with CT values >36 were chosen for further experimentation.

A laboratory strain of ACP collected from *Murraya paniculata* (L.) Jack (Aurantieae) at South China Agricultural University, Guangzhou, Guangdong, in 2013, was used in the present study. The strain had been maintained on *M. paniculata* in controlled environment chambers under a 14 h: 10 h light: dark cycle at 28 ± 1°C and 60% relative humidity for more than 25 generations.

Third to fourth instar psyllid nymphs were selected and reared on the *C*Las+ and uninfected (*C*Las–) ‘Shatangju’ plants for 14 days under a 14 h: 10 h light: dark cycle at 28 ± 1°C and 50–60% relative humidity. After this period, the heads of individual adult psyllids were dissected for DNA extraction using a Tiangen DNA extraction kit (Tiangen, Beijing, China) following which the *C*Las status of each individual was determined by qPCR as above.

### Transmission electron microscopy (TEM).

The abdomen of a *C*Las-infected ACP adult was dissected then fixed in 2.5% glutaraldehyde in 0.1 M phosphate-buffered saline (PBS; pH 7.2) at 4°C overnight. The tissue was then rinsed in 0.1 M PBS, secondarily fixed with 2% osmium tetroxide in 0.1 M PBS at room temperature for 4 h then embedded in Spurr’s resin. Sections (100 nm) of abdomen tissue were cut with a diamond knife, stained with 2% uranyl acetate and alkaline lead citrate. Sections were then examined using a transmission electron microscope (Advanced Microscopy Techniques Corp., Danvers, MA, United States) and photographed using a digital camera (Morgagni 268, FEI Company, Hillsboro, OR, United States).

### RNA extraction, RNA-seq and sequences assembly.

Two aliquots of 20 adult *C*Las+ and two aliquots 20 *C*Las– ACP were each pooled to constitute a sample to give two samples of each psyllid type and the thoraxes and abdomens ground in liquid nitrogen. RNA was extracted from each sample using TRIzol. Three biological replicates were made from every sample to give 12 extracts in total and used for RNA-seq. The resulting Illumina reads were trimmed using Trimmomatic v0.33 (87) to remove the adapters and low-quality sequences (Q ≤ 25). Trinity v2.13.0 (88) was then used for the *de novo* assembly of contigs from the trimmed RNA-seq reads. All data from each psyllid type were combined for analysis.

### Sequence analysis.

The reads and assembled contigs were preliminarily classified using the Kaiju virus database (89). Then, targeted contigs were subjected to a BLAST search (http://blast.ncbi.nlm.nih.gov/Blast.cgi). Viral sequences were aligned using MAFFT v7.471 with the l-INS-I strategy (90) and maximum likelihood (ML) trees were constructed using IQ-TREE 2.0 (91) to infer the phylogenetic relationships.

### Data availability.

All the RNA samples are deposited in −80°C refrigerator in the College of Natural Resource and Environment of China Agricultural University. And all the raw data and clean data of metatranscriptome sequencing are deposited in the private account of corresponding author of data storage center of College of Advanced Agricultural Science of Zhejiang A&F University. The newly generated viral sequences were deposited in https://github.com/ljmhaha/data.of.ACP.

## References

[1] Mendes R, Garbeva P, Raaijmakers JM. 2013. The rhizosphere microbiome: significance of plant beneficial, plant pathogenic, and human pathogenic microorganisms. FEMS Microbiol Rev 37:634–663. doi:10.1111/1574-6976.12028. 23790204

[2] Redman RS, Sheehan KB, Stout RG, Rodriguez RJ, Henson JM. 2022. Thermotolerance generated by plant/fungal symbiosis. Science 298:1581. doi:10.1126/science.1078055. 12446900

[3] Compant S, Reiter B, Sessitsch A, Nowak J, Clément C, Barka EA. 2005. Endophytic colonization of Vitis vinifera L. by plant growth-promoting bacterium Burkholderia sp. strain PsJN. Appl Environ Microbiol 71:1685–1693. doi:10.1128/AEM.71.4.1685-1693.2005. 15811990 PMC1082517

[4] Arnold AE, Mejía LC, Kyllo D, Rojas EI, Maynard Z, Robbins N, Herre EA. 2003. Fungal endophytes limit pathogen damage in a tropical tree. Proc Natl Acad Sci USA 100:15649–15654. doi:10.1073/pnas.2533483100. 14671327 PMC307622

[5] Susi H, Barrès B, Vale PF, Laine AL. 2015. Co-infection alters population dynamics of infectious disease. Nat Commun 6:1–8. doi:10.1038/ncomms6975. PMC4354079 25569306

[6] Saponari M, Loconsole G, Liao HH, Jiang B, Savino V, Yokomi RK. 2013. Validation of high-throughput real time polymerase chain reaction assays for simultaneous detection of invasive citrus pathogens. J Virol Methods 193:478–486. doi:10.1016/j.jviromet.2013.07.002. 23891873

[7] Borer ET, Laine AL, Seabloom EW. 2016. A multiscale approach to plant disease using the metacommunity concept. Annu Rev Phytopathol 54:397–418. doi:10.1146/annurev-phyto-080615-095959. 27296140

[8] Bomo LM, Orton RJ, Peredo EL, Morrison HG, Sistrom M, Simmons S, Turner PE. 2019. Spatiotemporal dynamics of RNA virus diversity in a phyllosphere microbial community. bioRxiv. doi:10.1101/772475.

[9] Wang XW, Blanc S. 2021a. Insect transmission of plant single-stranded DNA viruses. Annu Rev Entomol 66:21.1–21.17. doi:10.1146/annurev-ento-060920-094531. 32931313

[10] Xiang H-Y, Dong S-W, Shang Q-X, Zhou C-J, Li D-W, Yu J-L, Han C-G. 2011. Molecular characterization of two genotypes of a new polerovirus infecting brassicas in China. Arch Virol 156:2251–2255. doi:10.1007/s00705-011-1091-z. 21874520

[11] Lim S, Yoo RH, Igori D, Zhao F, Kim KH, Moon JS. 2015. Genome sequence of a recombinant brassica yellows virus infecting Chinese cabbage. Arch Virol 160:597–600. doi:10.1007/s00705-014-2258-1. 25352211

[12] Zhang XY, Xiang HY, Zhou CJ, Li DW, Yu JL, Han CG. 2014. Complete genome sequence analysis identifies a new genotype of brassica yellows virus that infects cabbage and radish in China. Arch Virol 159:2177–2180. doi:10.1007/s00705-014-2027-1. 24599564

[13] Yoshida N, Tamada T. 2019. Host range and molecular analysis of Beet leaf yellowing virus, Beet western yellows virus-JP and Brassica yellows virus in Japan. Plant Pathol 68:1045–1058. doi:10.1111/ppa.13023.

[14] Filardo F, Nancarrow N, Kehoe M, McTaggart AR, Congdon B, Kumari S, Aftab M, Trębicki P, Rodoni B, Thomas J, Sharman M. 2021. Genetic diversity and recombination between turnip yellows virus strains in Australia. Arch Virol 166:813–829. doi:10.1007/s00705-020-04931-w. 33481112

[15] Buxton-Kirk A, Adams I, Ward LFR, Kelly M, Forde S, Skelton A, Harju V, Baucas NS, BasIlan MAG, Joshi RC, Reeder RH, Annamalai S, Fox A, Fowkes A. 2021. First report of Turnip yellows virus in cabbage in the Philippines. New Dis Rep 44:e12020. doi:10.1002/ndr2.12020.

[16] Stevens M, Smith HG, Hallsworth PB. 1995. Detection of the luteoviruses, beet mild yellowing virus and beet western yellows virus, in aphids caught in sugar-beet and oilseed rape crops, 1990–1993. Ann Appl Biol 127:309–320. doi:10.1111/j.1744-7348.1995.tb06675.x.

[17] Schliephake E, Graichen K, Rabenstein F. 2000. Investigations on the vector transmission of the Beet mild yellowing virus (BMYV) and the Turnip yellows virus (TuYV). J Plant Dis Prot 107:81–87.

[18] Kaplan IB, Lee L, Ripoll DR, Palukaitis P, Gildow F, Gray SM. 2007. Point mutations in the potato leafroll virus major capsid protein alter virion stability and aphid transmission. J Gen Virol 88:1821–1830. doi:10.1099/vir.0.82837-0. 17485544

[19] Mulot M, Monsion B, Boissinot S, Rastegar M, Meyer S, Bochet N, Brault V. 2018. Transmission of turnip yellows virus by Myzus persicae is reduced by feeding aphids on double-stranded RNA targeting the ephrin receptor protein. Front Microbiol 9:457. doi:10.3389/fmicb.2018.00457. 29593696 PMC5859162

[20] Ghosh S, Kanakala S, Lebedev G, Kontsedalov S, Silverman D, Alon T, Mor N, Sela N, Luria N, Dombrovsky A, Mawassi M, Haviv S, Czosnek H, Ghanim M. 2019. Transmission of a new polerovirus infecting pepper by the whitefly Bemisia tabaci. J Virol 93:e00488-19. doi:10.1128/JVI.00488-19. 31092571 PMC6639281

[21] Patton MF, Bak A, Sayre JM, Heck ML, Casteel CL. 2020. A polerovirus, Potato leafroll virus, alters plant–vector interactions using three viral proteins. Plant Cell Environ 43:387–399. doi:10.1111/pce.13684. 31758809

[22] Collins JE, Benfield DA, Christianson WT, Harris L, Hennings JC, Shaw DP, Goyal SM, McCullough S, Morrison RB, Joo HS. 1992. Isolation of swine infertility and respiratory syndrome virus (isolate ATCC VR-2332) in North America and experimental reproduction of the disease in gnotobiotic pigs. J Vet Diagn Invest 4:117–126. doi:10.1177/104063879200400201. 1616975

[23] Chand RJ, Trible BR, Rowland RRR. 2012. Pathogenesis of porcine reproductive and respiratory syndrome virus. Curr Opin Virol 2:256–263. doi:10.1016/j.coviro.2012.02.002. 22709514

[24] Dea S, Gagnon CA, Mardassi H, Pirzadeh B, Rogan D. 2000. Current knowledge on the structural proteins of porcine reproductive and respiratory syndrome (PRRS) virus: comparison of the North American and European isolates. Arch Virol 145:659–688. doi:10.1007/s007050050662. 10893147 PMC7087215

[25] Nelsen CJ, Murtaugh MP, Faaberg KS. 1999. Porcine reproductive and respiratory syndrome virus comparison: divergent evolution on two continents. J Virol 73:270–280. doi:10.1128/JVI.73.1.270-280.1999. 9847330 PMC103831

[26] Valicek L, Psikal I, Smíd B, Rodák L, Kubalíková R, Kosinová E. 1997. Isolation and identification of porcine reproductive and respiratory syndrome virus in cell cultures. Vet Med 42:281–287. doi:10.1016/S0093-691X(97)00309-9. 9416008

[27] Tong G, Zhou YJ, Hao XF, Tian ZJ, An TQ, Qiu HJ. 2007. Highly pathogenic porcine reproductive and respiratory syndrome, China. Emerg Infect Dis 13:1434–1436. doi:10.3201/eid1309.070399. 18252136 PMC2857295

[28] Jiang Y, Li G, Yu L, Li L, Zhang Y, Zhou Y, Tong W, Liu C, Gao F, Tong G. 2020. Genetic diversity of porcine reproductive and respiratory syndrome virus (PRRSV) from 1996 to 2017 in China. Front Microbiol 11:618. doi:10.3389/fmicb.2020.00618. 32390968 PMC7193098

[29] Yu F, Yan Y, Shi M, Liu HZ, Zhang HL, Yang YB, Huang XY, Gauger PC, Zhang JQ, Zhang YH, Tong GZ, Tian ZJ, Chen JJ, Cai XH, Liu D, Li GW, An TQ. 2020. Phylogenetics, genomic recombination, and NSP2 polymorphic patterns of porcine reproductive and respiratory syndrome virus in China and the United States in 2014–2018. J Virol 94:e01813. doi:10.1128/JVI.01813-19. 31896589 PMC7158704

[30] Reiner G, Fresen C, Bronnert S, Willems H. 2009. Porcine Reproductive and Respiratory Syndrome Virus (PRRSV) infection in wild boars. Vet Microbiol 136:250–258. 30. doi:10.1016/j.vetmic.2008.11.023. 19131188

[31] Otake S, Dee SA, Rossow KD, Moon RD, Pijoan C. 2002. Mechanical transmission of porcine reproductive and respiratory syndrome virus by mosquitoes, Aedes vexans (Meigen). Can J Vet Res 66:191–195. 12146891 PMC227003

[32] Otake S, Dee SA, Moon RD, Rossow KD, Trincado C, Pijoan C. 2003. Evaluation of mosquitoes, Aedes vexans, as biological vectors of porcine reproductive and respiratory syndrome virus. Can J Vet Res 67:265–270. 14620862 PMC280710

[33] Schurrer JA, Dee SA, Moon RD, Murtaugh MP, Finnegan CP, Deen J, Kleiboeker SB, Pijoan CBJ. 2005. Retention of ingested porcine reproductive and respiratory syndrome virus in houseflies. Am J Vet Res 66:1517–1525. doi:10.2460/ajvr.2005.66.1517. 16261824

[34] Pitkin A, Deen J, Otake S, Moon R, Dee R. 2009. Further assessment of houseflies (Musca domestica) as vectors for the mechanical transport and transmission of porcine reproductive and respiratory syndrome virus under field conditions. Can J Vet Res 73:91–96. 19436589 PMC2666325

[35] Bové JM. 2006. Huanglongbing: a destructive, newly-emerging, century-old disease of citrus. J Plant Pathol 88:7–37. doi:10.4454/jpp.v88i1.828.

[36] Hajeri S, Killiny N, El-Mohtar C, Dawson WO, Gowda S. 2014. Citrus tristeza virus-based RNAi in citrus plants induces gene silencing in Diaphorina citri, a phloem-sap sucking insect vector of citrus greening disease (Huanglongbing). J Biotechnol 176:42–49. doi:10.1016/j.jbiotec.2014.02.010. 24572372

[37] Matsumura EE, Nerva L, Nigg JC, Falk BW, Nouri S. 2016. Complete genome sequence of the largest known flavi-like virus, Diaphorina citri flavi-like virus, a novel virus of the Asian citrus psyllid, Diaphorina citri. Genome Announc 4:e00946-16. doi:10.1128/genomeA.00946-16. 27609921 PMC5017226

[38] Nigg JC, Nouri S, Falk BW. 2016. Complete genome sequence of a putative densovirus of the Asian citrus psyllid, Diaphorina citri. Genome Announc 4:e00589. doi:10.1128/genomeA.00589-16. 27469948 PMC4966452

[39] Nouri S, Salem N, Nigg JC, Falk BW. 2016. Diverse array of new viral sequences identified in worldwide populations of the Asian citrus psyllid (Diaphorina citri) using viral metagenomics. J Virol 90:2434–2445. doi:10.1128/JVI.02793-15. PMC4810699 26676774

[40] Britt K, Gebben S, Levy A, Rwahnih MA, Batuman O. 2020. The detection and surveillance of Asian Citrus Psyllid (Diaphorina citri)—associated viruses in Florida citrus groves. Front Plant Sci 10:1687. doi:10.3389/fpls.2019.01687. 32010169 PMC6978739

[41] Nigg JC, Falk BW. 2020. Diaphorina citri densovirus is a persistently infecting virus with a hybrid genome organization and unique transcription strategy. J Gen Virol 101:226–239. doi:10.1099/jgv.0.001371. 31855134

[42] Chen YM, Sadiq S, Tian JH, Chen X, Lin XD, Shen JJ, Chen H, Hao ZY, Wille M, Zhou ZC, Wu J, Li F, Wang HW, Yang WD, Xu QY, Wang W, Gao WH, Holmes EC, Zhang YZ. 2022. RNA viromes from terrestrial sites across China expand environmental viral diversity. Nat Microbiol 7:1312–1323. doi:10.1038/s41564-022-01180-2. 35902778

[43] Maclot F, Candresse T, Filloux D, Malmstrom CM, Roumagnac R, van der Vlugt R, Massart S. 2020. Illuminating an ecological blackbox: using high throughput sequencing to characterize the plant virome across scales. Front Microbiol 11:578064. doi:10.3389/fmicb.2020.578064. 33178159 PMC7596190

[44] Amimo JO, El Zowalaty ME, Githae D, Wamalwa M, Djikeng A, Nasrallah GK. 2016. Metagenomic analysis demonstrates the diversity of the fecal virome in asymptomatic pigs in East Africa. Arch Virol 161:887–897. doi:10.1007/s00705-016-2819-6. 26965436

[45] Wang Q, Xu FZ, An LL, Zhang WH. 2019. Molecular characterization of a new recombinant brassica yellows virus infecting tobacco in China. Virus Genes 55:253–256. doi:10.1007/s11262-019-01636-4. 30697673

[46] He C, Zhao X, Fan LJ, Li SF, Wang HQ. 2022. Strawberry, a new natural host of brassica yellows virus in China. Plant Dis 106:1079. doi:10.1094/PDIS-08-21-1617-PDN.34546782

[47] Gnanasekaran P, Chakraborty S. 2018. Biology of viral satellites and their role in pathogenesis. Curr Opin Virol 33:96–105. doi:10.1016/j.coviro.2018.08.002. 30144641

[48] Gui X, Liu C, Qi Y, Zhou X. 2022. Geminiviruses employ host DNA glycosylases to subvert DNA methylation-mediated defense. Nat Commun 13:1–11. doi:10.1038/s41467-022-28262-3. 35102164 PMC8803994

[49] Jiang GY, Wang C, Song L, Wang X, Zhou YY, Fei CN, Liu H. 2021. Aerosol transmission, an indispensable route of COVID-19 spread: case study of a department-store cluster. Front Environ Sci Eng 15:46. doi:10.1007/s11783-021-1386-6. 33391845 PMC7771204

[50] Wang B, Tang Z, Cai N, Niu H. 2021b. The characteristics and sources apportionment of water-soluble ions of PM_2.5_ in suburb Tangshan, China. Urban Clim 35:100742. doi:10.1016/j.uclim.2020.100742.

[51] Ji B, Zhao Y, Esteve-Núñez A, Liu R, Yang Y, Nzihou A, Tai Y, Wei T, Shen C, Yang Y, Ren B, Wang X, Wang Y. 2021. Where do we stand to oversee the coronaviruses in aqueous and aerosol environment? characteristics of transmission and possible curb strategies. Chem Eng J 413:127522. doi:10.1016/j.cej.2020.127522. 33132743 PMC7590645

[52] Xu L, Zhang J, Sun X, Xu S, Shan M, Yuan Q, Liu L, Du Z, Liu D, Xu D, Song C, Liu B, Lu G, Shi Z, Li W. 2020. Variation in Concentration and Sources of Black Carbon in a Megacity of China During the COVID-19 Pandemic. Geophys Res Lett 47:e2020GL090444. doi:10.1029/2020GL090444. PMC7744912 33349736

[53] Philippot L, Raaijmakers JM, Lemanceau P, van der Putten WH. 2013. Going back to the roots: the microbial ecology of the rhizosphere. Nat Rev Microbiol 11:789–799. doi:10.1038/nrmicro3109. 24056930

[54] Campbell RN. 1996. Fungal transmission of plant viruses. Annu Rev Phytopathol 34:87–108. doi:10.1146/annurev.phyto.34.1.87. 15012536

[55] Xu J, Zhang Y, Zhang P, Trivedi P, Riera N, Wang Y, Liu X, Fan G, Tang J, Coletta-Filho HD, Cubero J, Deng X, Ancona V, Lu Z, Zhong B, Roper MC, Capote N, Catara V, Pietersen G, Vernière C, Al-Sadi AM, Li L, Yang F, Xu X, Wang J, Yang H, Jin T, Wang N. 2018. The structure and function of the global citrus root-associated microbiome. Nat Commun 9:4894. doi:10.1038/s41467-018-07343-2. 30459421 PMC6244077

[56] Hiruma K, Gerlach N, Sacristán S, Nakano RT, Hacquard S, Kracher B, Neumann U, Ramírez D, Bucher M, O’Connell RJ. 2016. Root endophyte Colletotrichum tofieldiae confers plant fitness benefits that are phosphate status dependent. Cell 165:464–474. doi:10.1016/j.cell.2016.02.028. 26997485 PMC4826447

[57] Zuo DP, He MJ, Chen XR, Hu RJ, Zhao TY, Zhang XY, Peng YM, Wang Y, Li DW, Yu JL, Han CG. 2021. A simple method for the acquisition and transmission of brassica yellows virus from transgenic plants and frozen infected leaves by aphids. Plants 10:1944. doi:10.20944/preprints202008.0232.v1. 34579476 PMC8471377

[58] Chen DM, Chen WM, Chen SS. 1993. The succession of the predominant citrus aphids and the control of pesticides. Acta Agriculturae Zhejiangensis 5:42–45. (In Chinese).

[59] Fujisawa I. 1985. Aphid transmission of turnip mosaic virus and cucumber mosaic virus 2 transmission from virus mixtures. Ann Phytopathol Soc Jpn 51:562–568. doi:10.3186/jjphytopath.51.562.

[60] Blackman RL, Eastop VF. 1994. Aphids on the world's trees: an identification and information guide. CAB International.

[61] Owolabi AT, Ekpiken EE. 2014. Transmission efficiency of two strains of Moroccan watermelon mosaic virus by two clones of Aphis spiraecola (Patch). Int J Virol 10:253–262. https://scialert.net/abstract/?doi=ijv.2014.253.262. doi:10.3923/ijv.2014.253.262.

[62] van Emden HF, Eastop VF, Hughes RD, Way MJ. 1969. The ecology of Myzus persicae. Annu Rev Entomol 14:197–270. doi:10.1146/annurev.en.14.010169.001213.

[63] Troncoso AJ, Vargas RR, Tapia DH, Olivares-Donoso R, Niemeyer HM. 2005. Host selection by the generalist aphid Myzus persicae (Hemiptera: Aphididae) and its subspecies specialized on tobacco, after being reared on the same host. B Entomol Res 95:23–28. doi:10.1079/BER2004334. 15705211

[64] Pan LL, Chen QF, Guo T, Wang XR, Li P, Wang XW, Liu SS. 2018. Differential efficiency of a begomovirus to cross the midgut of different species of whiteflies results in variation of virus transmission by the vectors. Sci China Life Sci 61:1254–1265. doi:10.1007/s11427-017-9283-4. 29785572

[65] Mei Y, Wang Y, Hu T, He ZF, Zhou XP. 2021. The C4 protein encoded by tomato leaf curl Yunnan virus interferes with mitogen-activated protein kinase cascade-related defense responses through inhibiting the dissociation of the ERECTA/BKI1 complex. New Phytol 231:747–762. doi:10.1111/nph.17387. 33829507

[66] Wang MM. 2018. Identification and genetic transformation of citrus MAPK genes and PR genes induced by Huanglongbing. Master’s Thesis, Southwest University, China (In Chinese).

[67] Lennon JT, Lehmkuhl BK. 2016. A trait-based approach to bacterial biofilms in soil. Environ Microbiol 18:2732–2742. doi:10.1111/1462-2920.13331. 27104876

[68] Storey MV, Ashbolt NJ. 2001. Persistence of two model enteric viruses (B40-8 and MS-2 bacteriophages) in water distribution pipe biofilms. Water Sci Technol 43:133–138. . doi:10.2166/wst.2001.0724. 11464741

[69] Nath S, Aron GM, Southard GM, McLean RJC. 2010. Potential for largemouth bass virus to associate with and gain protection from bacterial biofilms. J Aquat Anim Health 22:95–101. doi:10.1577/H09-046.1. 20848884

[70] Ha PT, He R, Killiny N, Brown JK, Omsland A, Gang DR, Beyenal H. 2019. Host-free biofilm culture of “Candidatus Liberibacter asiaticus,” the bacterium associated with Huanglongbing. Biofilm 1:100005. doi:10.1016/j.bioflm.2019.100005. 33447792 PMC7798463

[71] Wang N, Stelinski LL, Pelz-Stelinski KS, Graham JH, Zhang Y. 2017. Tale of the huanglongbing disease pyramid in the context of the citrus microbiome. Phytopathology 107:380–387. doi:10.1094/PHYTO-12-16-0426-RVW. 28095208

[72] Tian L, Wang L. 2021. Multi-omics analysis reveals structure and function of biofilm microbial communities in a pre-denitrification biofilter. Sci Total Environ 757:143908. doi:10.1016/j.scitotenv.2020.143908. 33316516

[73] Yi WY, Shen JL, Liu GP, Wang J, Yu LF, Li Y, Peis S, Wu JS. 2021. High NH3 deposition in the environs of a commercial fattening pig farm in central south China. Environ Res Lett 16:125007. doi:10.1088/1748-9326/ac3603.

[74] Wang HC, Feng ZH, Peng L, Li MY. 2011. Influence of environmental factors on the co-aggregation ability of Bacillus megaterium T1 as bridging bacterium. J Ah Agr Sci 39:9791–9792. doi:10.3969/j.issn.0517-6611.2011.16.128.

[75] Meehl GA, Senior CA, Eyring V, Flato G, Lamargue J, Stouffer RJ, Taylor KE, Schlund M. 2020. Context for interpreting equilibrium climate sensitivity and transient climate response from the CMIP6 Earth system models. Sci Adv 6:eaba1981. doi:10.1126/sciadv.aba1981. 32637602 PMC7314520

[76] Yi YC, Shen JL, Yang CD, Wang J, Li Y, Wu JS. 2020. Dry deposition of ammonia around paddy fields in the subtropical hilly area in southern China. Atmos Ocean Sci Lett 13:216–223. doi:10.1080/16742834.2020.1738208.

[77] Dwiastuti ME. 2020. Citrus foot rot disease (Phytophthora spp.) control in Indonesia using good agricultural practices efforts green agroindustry. IOP Conf Ser: Earth Environ Sci 484:e012097. doi:10.1088/1755-1315/484/1/012097.

[78] Rusiñol M, Hundesa A, Cárdenas-Youngs Y, Fernández-Bravo A, Pérez-Cataluña A, Moreno-Mesonero L, Moreno Y, Calvo M, Alonso JL, Figueras MJ, Araujo R, Bofill-Mas S, Girones R. 2020. Microbiological contamination of conventional and reclaimed irrigation water: evaluation and management measures. Sci Total Environ 710:136298. doi:10.1016/j.scitotenv.2019.136298. 31923670

[79] Zhao M, Liu R, Wang X, Zhang J, Wang J, Cao B, Zhao Y, Xu L, Chen Y, Zou G. 2022. How do controlled-release fertilizer coated microplastics dynamically affect Cd availability by regulating Fe species and DOC content in soil? Sci Total Environ 850:157886. doi:10.1016/j.scitotenv.2022.157886. 35952884

[80] Lanver D, Tollot M, Schweizer G, Presti LL, Reissmann S, Ma LS, Schuster M, Tanaka S, Liang L, Ludwig N, Kahmann R. 2017. Ustilago maydis effectors and their impact on virulence. Nat Rev Microbiol 15:409–421. doi:10.1038/nrmicro.2017.33. 28479603

[81] Clark K, Franco JY, Schwizer S, Pang ZQ, Hawara E, Liebrand TWH, Pagliaccia D, Zeng L, Gurung FB, Wang PC, Shi JX, Wang YS, Ancona V, van der Hoorn RAL, Wang N, Coaker G, Ma WB. 2018. An effector from the Huanglongbing-associated pathogen targets citrus proteases. Nat Commun 9:1718. doi:10.1038/s41467-018-04140-9. 29712915 PMC5928222

[82] Britt K, Gebben S, Levy A, Achor D, Sieburth P, Stevens K, Rwahnih M, Batuman O. 2022. Analysis of citrus tristeza virus incidences within Asian citrus psyllid (Diaphorina citri) populations in Florida via high-throughput sequencing. Insects 13:275. doi:10.3390/insects13030275. 35323573 PMC8954720

[83] Folimonova SY, Robertson CJ, Garnsey SM, Gowda S, Dawson WO. 2009. Examination of the responses of different genotypes of citrus to huanglongbing (citrus greening) under different conditions. Phytopathology 99:1346–1354. doi:10.1094/PHYTO-99-12-1346. 19900000

[84] Fu S, Shao J, Paul C, Zhou C, Hartung JS. 2017. Transcriptional analysis of sweet orange trees co-infected with ‘Candidatus Liberibacter asiaticus’ and mild or severe strains of Citrus tristeza virus. BMC Genomics 18:1–7. doi:10.1186/s12864-017-4174-8. 29089035 PMC5664567

[85] van Vuuren SP, van der Vyver JB, Luttig M, da Graça JV. 2000. Low incidence of Huanglongbing fruit symptoms in Valencia sweet orange trees in the presence of a population of Citrus tristeza virus. International Organization of Citrus Virologists Conference Proceedings (1957–2010) 14(14): 373–377.

[86] Li WB, Hartung JS, Levy L. 2006. Quantitative real-time PCR for detection and identification of “Candidatus Liberibacter” species associated with citrus Huanglongbing. J Microbiol Methods 66:104–115. doi:10.1016/j.mimet.2005.10.018. 16414133

[87] Bolger AM, Lohse M, Usadel B. 2014. Trimmomatic: a flexible trimmer for Illumina sequence data. Bioinformatics 30:2114–2120. doi:10.1093/bioinformatics/btu170. 24695404 PMC4103590

[88] Grabherr MG, Haas BJ, Yassour M, Levin JZ, Thompsone DA, Amit I, Adiconis X, Fan L, Raychowdhury R, Zeng Q, Chen Z, Mauceli E, Hacohen N, Gnirke A, Rhind N, di Palma F, Birren BW, Nusbaum C, Lindblad-Toh K, Friedman N, Regev A. 2011. Trinity: reconstructing a full-length transcriptome without a genome from RNA-Seq data. Nat Biotechnol 29:644–652. doi:10.1038/nbt.1883. 21572440 PMC3571712

[89] Menzel P. 2016. Fast and sensitive taxonomic classification for metagenomics with Kaiju. Nat Commun 7:1–9. doi:10.1038/ncomms11257. PMC4833860 27071849

[90] Katoh K, Standley DM. 2013. MAFFT multiple sequence alignment software version 7: improvements in performance and usability. Mol Biol Evol 30:772–780. doi:10.1093/molbev/mst010. 23329690 PMC3603318

[91] Minh BQ, Schmidt HA, Chernomor O, Schrempf D, Woodhams MD, von Haeseler A, Lanfear R. 2020. IQ-TREE 2: new models and efficient methods for phylogenetic inference in the genomic era. Mol Biol Evol 37:1530–1534. doi:10.1093/molbev/msaa015. 32011700 PMC7182206

